# The Role of miRNA-7 in the Biology of Cancer and Modulation of Drug Resistance

**DOI:** 10.3390/ph14020149

**Published:** 2021-02-12

**Authors:** Ewa Gajda, Małgorzata Grzanka, Marlena Godlewska, Damian Gawel

**Affiliations:** 1 Department of Biochemistry and Molecular Biology, Centre of Postgraduate Medical Education, Marymoncka 99/103, 01-813 Warsaw, Poland; ewa.gajda@cmkp.edu.pl (E.G.); marlena.godlewska@cmkp.edu.pl (M.G.); 2 Department of Immunohematology, Centre of Postgraduate Medical Education, Marymoncka 99/103, 01-813 Warsaw, Poland

**Keywords:** miR-7, multidrug resistance, cancer

## Abstract

MicroRNAs (miRNAs, miRs) are small non-coding RNA (ncRNA) molecules capable of regulating post-transcriptional gene expression. Imbalances in the miRNA network have been associated with the development of many pathological conditions and diseases, including cancer. Recently, miRNAs have also been linked to the phenomenon of multidrug resistance (MDR). MiR-7 is one of the extensively studied miRNAs and its role in cancer progression and MDR modulation has been highlighted. MiR-7 is engaged in multiple cellular pathways and acts as a tumor suppressor in the majority of human neoplasia. Its depletion limits the effectiveness of anti-cancer therapies, while its restoration sensitizes cells to the administered drugs. Therefore, miR-7 might be considered as a potential adjuvant agent, which can increase the efficiency of standard chemotherapeutics.

## 1. Introduction

The first miRNA (miRNA, miR) was discovered separately by Ambros and Ruvkun in 1993 [1,2]. Nowadays, nearly 2300 miRs have been recognized. Around 50% of miR sequences are accessible in online databases (miRbase; http://www.mirbase.org/; miRBase V22) and multiple software tools allow to predict targets for miRs of choice [3].

**Figure 1:**
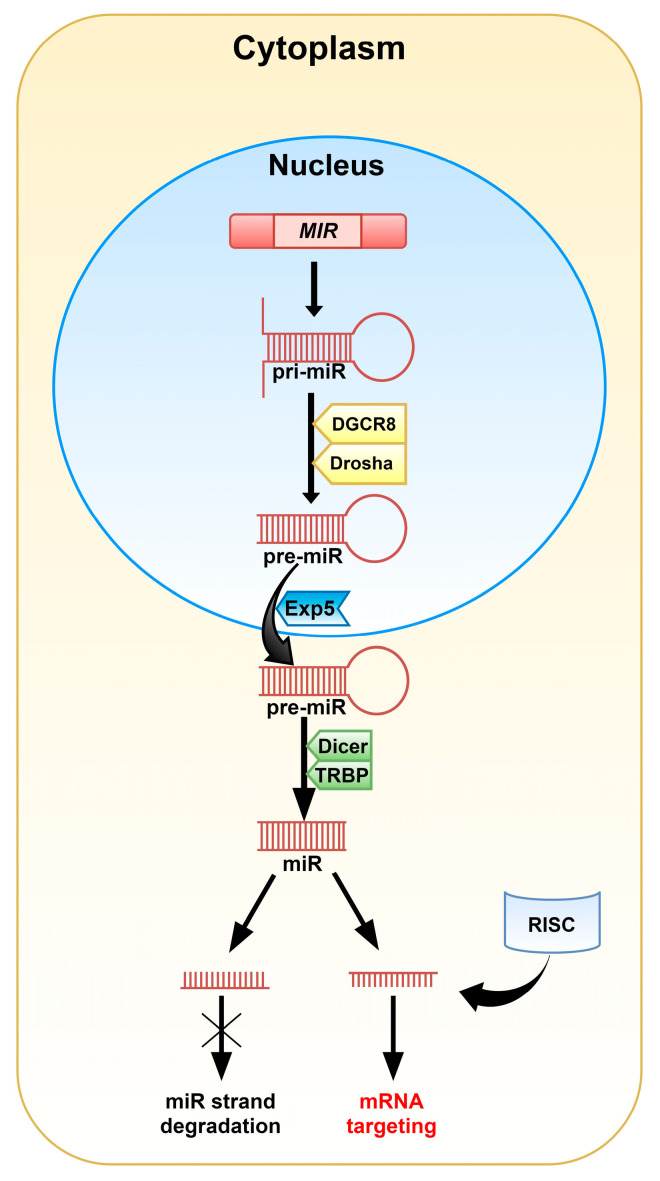
A graphical representation of the canonical pathway of miRNA biogenesis in animals. The *MIR* gene encodes pri-miRNA. Modification of the pri-miRNA hairpin with two free ends takes place in the cell nucleus. The DGCR8 (DiGeorge critical region 8) and Drosha enzymes cut off the free strands from the hairpin giving pre-miRNA. Then, pre-miRNA is transported into the cytoplasm by Exportin 5 (Exp5), where the Dicer and TRBP (TAR double-stranded RNA binding protein) complex removes the hairpin’s loop and cleaves the molecule into the miRNA duplex. One of the duplex strands, along with the RNA-induced silencing complex (RISC), is involved in mRNA targeting. The second one is degraded.

MiRNAs are 20-22 nucleotide-long non-coding RNAs (ncRNAs) that play a crucial role in post-transcriptional regulation of gene expression and their biogenesis is well established. In most cases, miRs are synthesized via the canonical pathway. In this mode, miRs are transcribed individually or as a polycistronic transcript by RNA polymerase II (Pol II), less often by RNA polymerase III (Pol III), in a multistep process. The transcription of miRs is initiated by the formation of primary miRNA (pri-miRNA; Figure 1) in the shape of a hairpin [4,5,6]. The microprocessor complex, containing among others, the DiGeorge critical region 8 (DGCR8) and Drosha enzymes, identifies specific motifs within the sequence of pri-miRNA and releases precursor miRNA (pre-miRNA) by cleaving the stem of the hairpin. This process takes place in the nucleus, but once pre-miRNA is formed, Exportin 5 (Exp5) facilitates its transport to the cytoplasm. The complex made of the Dicer enzyme and TRBP (TAR double-stranded RNA binding protein) captures shuttled pre-miRNA. Dicer presents ribonuclease activity and cuts off the loop of the hairpin and cleaves long double-stranded RNA (dsRNA) into shorter ~20 nucleotide fragments. The generated single-stranded mature miRNA originated from the 5′ and 3′ strands are called 5p and 3p, respectively.

Biogenesis of miRs via the non-canonical pathways has also been described and is generally divided into two groups: Dicer- and Drosha/DGCR8-independent paths. This mode is often linked with pathological conditions, including cancer [4,7].

The miRNA guide strand anchored to the Argonaute 2 (Ago2) protein targets mRNA during the RNA-induced silencing complex (RISC) loading process [4,5]. Mature miR contains a 2-8 nucleotide-long “seed sequence” that binds to target nucleotides in the 3′ untranslated region of cognate mRNAs, however, binding sites have also been reported in the 5′UTR and coding sequence. The rest of the miR sequence can bind with less complementarity and this allows a miRNA to target multiple mRNAs [4].

The formation of miRNA/Ago2 can harm the production of peptides on various levels, such as: induction of mRNA degradation, affecting proper ribosome assembly and finally, degradation of growing peptides during the process of translation. While the degradation of the mRNA sequence is permanent, the repression of translation can be reversed through detaching miRNA [6]. It has been shown that one microRNA can bind to hundreds of nearly-complementary mRNA sequences and also, one mRNA might be a target for several miRs [8]. MiRs may also cooperate with other ncRNAs including long non-coding RNAs (lncRNAs) [9]. It is assumed that miRs control the expression of nearly 60% of protein-coding genes [10].

MiRNAs are crucial for maintaining cellular development [11,12], differentiation [12,13,14], the cell cycle [15,16,17], proliferation [18,19,20,21], migration [22,23], and apoptosis [24,25]. Apart from their intracellular location, miRNAs are found in biological fluids, such as plasma, saliva, urine, breast milk and might be transferred from one species to another [10,26]. It has been observed that disruption of the miRNA network profile can be linked to several diseases, including cardiovascular diseases, nervous system disorders, and sepsis. Imbalance in the miRNA pool has also been reported in various tumors, such as brain, breast, lung, and colon cancer. MiRNAs may act as either cancer suppressors or as oncogenic factors (oncomiRs). MiR-17-92, miR-21, miR-106, and miR-191 are involved in the development of cancer. Their increased expression has been observed in lung, breast, and gastric cancer, as well as in glioblastoma (GB). On the other hand, depletion of miR-15a, miR-34a, and/or miR-126 suppresses the progression of lung, prostate, and breast tumors [10]. Often, the role of the defined miRNAs is tissue-specific. For example, miR-24 and miR-221/222 are recognized as oncomiRs in breast cancer and glioblastoma, while they act as suppressors in laryngeal or tongue squamous cell carcinoma. Similarly, a dual role of miRNA was observed for miR-155 and miR-125. Lack of miRNA homeostasis in cancer cells promotes enhanced proliferation, angiogenesis, migration, and invasiveness, while blocking apoptosis [27].

It is considered that miRNA can also play a key role in triggering multidrug resistance (MDR) in cancer cells. MDR is a rising therapeutic problem in the treatment of numerous types of tumors as it significantly decreases the effectiveness of anti-cancer drug therapies. Various mechanisms are involved in MDR, including induction of anti-apoptotic machinery or overexpression/activation of several ATP binding cassette (ABC) transporters [28]. Among the 49 ABC proteins, P-glycoprotein (P-gp/*ABCB1*), breast cancer resistance protein (BCRP/*ABCG2*), and multidrug resistance-associated protein 1 (MRP1/*ABCC1*) are the most studied. It has been shown that their high expression correlates with poor prognosis in cancer patients and that a significant portion of cancer-related deaths might be linked with MDR [29,30,31,32,33]. Therefore, there is a need for (i) better understanding of how expression and activity of MDR proteins is managed in tumor cells, (ii) identification of critical genes/proteins/pathways involved in the MDR phenotype of cancer cells, and (iii) elucidation of the role of miRNA in modulation of the MDR phenomenon. Application of antibody- and nano-based vehicles has resulted in major progress in the development of innovative drug delivery systems over the last years [34,35]. Convergence of these strategies with miRNA’s properties might significantly improve therapeutic procedures and effectively impair the progression of diseases, especially in terms of escaping MDR action. The role of miRNA-7 in carcinogenesis and modulation of MDR-encoding genes has been especially highlighted and studied.

## 2. MiRNA-7

**Figure 2:**
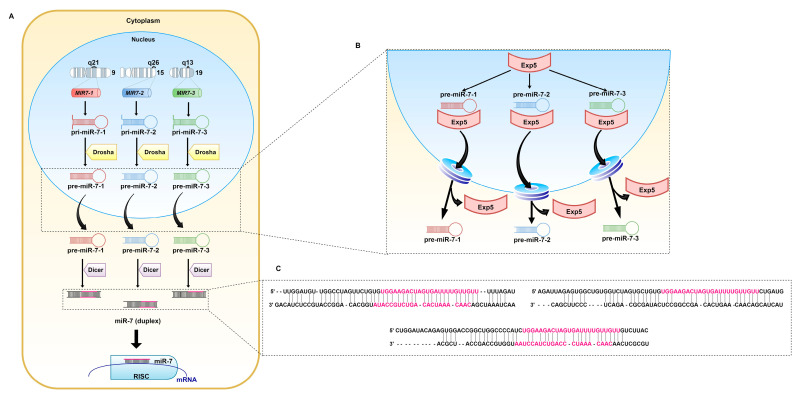
Biogenesis of miR-7. (**A**) miR-7 is the result of transcription of the *MIR7-1*, *MIR7-2*, and *MIR7-3* genes located on chromosomes 9, 15, and 19, respectively. The generated pri-miRs are transformed into pre-miR-7. Exp5 transports them through the nuclear pores to the cytoplasm (**B**), where pre-miR-7-1, pre-miR-7-2, and pre-miR-7-3 (**C**) undergo further modifications. The Dicer complex, cleaves double-stranded RNA (dsRNA) into shorter nucleotide duplexes. Sequences marked in pink are involved in the regulation of mRNA expression.

MiRNA-7 (miR-7, hsa-miRNA-7) was first reported in *Drosophila melanogaster*, nevertheless, the sequence of the guide strand is strongly conserved across different species, which highlights its importance [36]. In humans, miR-7 originates from three precursors: pri-miR-7-1, pri-miR-7-2, and pri-miR-7-3 (Figure 2). They are encoded by the *MIR7-1*, *MIR7-2*, and *MIR7-3* genes located on three chromosomes: 9q21, 15q26, and 19q13, respectively [37]. Pri-miR-7-1 and pri-miR-7-3 lie within introns of the heterogeneous nuclear ribonucleoprotein K-encoding gene (*HNRNPK*) and pituitary specific factor 1 gene (*PIT1*), respectively. The pri-miR-7-2-encoding gene is placed in the intergenic region of chromosome 15 [38,39]. Originally, miR-7 referred to miR-7-5p, since it seemed to be the only mature miRNA from all three precursors that affects cellular pathways. However, other biologically significant miRNAs, miR-7-1-3p and miRNA-7-2-3p, have also been reported [40,41,42,43,44,45]. There are slight changes within the sequence of nucleotides between the miRNAs.

### 2.1. MiR-7 Biogenesis and Regulation

Mature miR-7 is the product of the canonically transcribed *MIR7-1*, *MIR7-2*, and *MIR7-3* genes and regulation of transcription proceeds separately for each locus.

Apart from regulation of expression of genes encoding for proteins engaged in maturation of miRNA, which applies to all miRNAs, the expression of miR-7 is additionally regulated at the transcriptional level via transcription factors binding to their promoters (Table 1) [36].

It has been shown that transcription of *MIR7-1* may be activated by c-Myc [46] and homeobox D10 (HOXD10) [47], whereas hepatocyte nuclear factor 4 alpha (HNF4α) induces *MIR7-2* transcription [48]. Another transcriptional factor capable of enhancing miR-7 expression is forkhead box P3 (FOXP3) [49]. An opposite role in miR-7 regulation was revealed for v-Rel avian reticuloendoheliosis viral oncogene homolog A (RELA), which has a binding site in the promotor region of *MIR7-1* and *MIR7-2.* MiR-7 itself also inhibits RELA in a negative feedback manner, by directly binding to the 3′UTR of the RELA transcript [50,51,52]. Ubiquitin-specific protease 18 (Usp18) is another transcription inhibitor of all *MIR7.* Usp18 decreases the expression of miR-7 host genes, as well as intergenic pri-miR-7-2 [53].

MiR-7 biogenesis is also regulated at the post-transcriptional level. The product of *MIR7-1* (pri-miR-7-1) might undergo regulation by the Hu antigen R (HuR) and the Musashi homolog 2 (MSI2) complex. HuR enhances MSI2 binding to the pri-miR-7 conserved terminal loop. This inhibits maturation of pri-miR-7-1 to pre-miR-7-1 [54]. The QKI-5 and QKI-6 proteins restrain miR-7 biogenesis from the *MIR7-1* gene. Both proteins directly bind to the pri-miR-7-1 sequence, preventing its further processing and capturing the transcript within the nucleus [55]. The SF2/ASF splicing factor directly connects to pri-miR-7 and supports Drosha in cleavage and maturation [56].

Lastly, mature miR-7 can be modulated by competitive endogenous RNAs (ceRNAs) such as circular RNAs (circRNAs) [57]. They are highly stable and resistant to exonuclease activity due to their covalently closed loop form [58]. The first documented circRNA attenuating miR-7 was derived from the *CDR1* gene antisense strand and it is known as ciRS-7 (CDR1as). CiRS-7 is highly expressed in brain and neuronal tissue and contains over 70 seed-matched binding sites for miR-7. It may abate silencing of miR-7 targeted transcripts in brain, non-small cell lung cancer (NSCLC), esophageal squamous cell carcinoma, papillary thyroid cancer, and colorectal cancer [59,60,61,62,63]. CircSNCA is another circular RNA inhibiting miR-7 in brain and neuronal tissue. The treatment-induced muting of circSNCA alters the miR-7 level and induces apoptosis and autophagy in Parkinson’s disease [64]. Gao et al. (2017, 2019), based on microarray analysis, discovered that expression of circ_0006528 is elevated in doxorubicin (DOX, adriamycin)-resistant breast cancer MCF-7 cells and tumor tissues. It was confirmed that circ_0006528 silencing increases expression of miR-7-5p [65,66]. Additionally, Li et al. (2019) indicated that circ-U2AF1 (circ_0061868) also presents direct binding properties to miR-7-5p. *U2AF1* encodes for the U2 small nuclear RNA auxiliary factor 1 protein and plays a significant role in RNA splicing as part of the U2 auxiliary complex. The level of circ-U2AF1 is increased in glioma tissues. It was found that downregulation of circ-U2AF1 results in upregulation of miR-7-5p [67]. Until now, researchers have discovered several novel circRNAs working as a miR-7 sponge, such as circ_0000735 in prostate cancer [68], circ-ITCH in osteosarcoma [69], circ-TFCP2L1 in breast cancer [70], and circ_0015756 in hepatocellular carcinoma (HCC) [71].

LncRNAs are an alternative group of ncRNAs that play a significant role in regulating the network of gene expression and miRNAs [72]. Recently, altered expression of lncRNAs was linked with miR-7 silencing in multiple tumors. Zheng et al. (2020) confirmed competitive binding properties of cancer susceptibility 21 (CASC21) lncRNA to miR-7-5p, which results in activation of YAP1 in colorectal cancer [73]. Other lncRNAs targeting miR-7-5p in colorectal cancer are lncRNA RP4, terminal differentiation-induced non-coding RNA (TINCR), and Rhophilin Rho GTPase binding protein 1 antisense RNA 1 (RHPN1-AS1 lcRNA) [74,75,76]. Song et al. (2020) confirmed the effect of RHPN1-AS1 lcRNA in HCC [77]. KCNQ1 overlapping transcript 1 (KCNQ1OT1) lncRNA, which interacts with miR-7-5p, is an example of a lncRNA-based MDR modulator in HCC [78].

It has been reported that lncRNA LINC00115, which is upregulated in triple-negative breast cancer and correlates with poor prognosis, acts as a sponge of miR-7-5p [79]. LINC00115 may also regulate miR-7 expression in lung adenocarcinoma [80]. In renal cell carcinoma, maternally expressed gene 3 (MEG3) lncRNA, which is overexpressed in this type of cancer, targets miR-7 [81]. The activity of MEG3 is frequently decreased in various tumors, such as HCC, cervical, and breast cancer. Another lncRNA found in breast cancer is the Hox transcript antisense intergenic RNA (HOTAIR), which suppresses miR-7 indirectly [82].

FOXD2 adjacent opposite strand RNA 1 (FOXD2-AS1) is an oncogenic lncRNA. Thyroid cancer survivors with high expression of FOXD2-AS1 are more prone to relapse. Liu et al. (2019) proved that FOXD2-AS1 has a binding site for miR-7-5p and its downregulation restores a decreased level of miR-7-5p in thyroid carcinoma [83].

Other lncRNAs affecting miR-7-5p include SOX21 antisense RNA 1 (SOX21-AS1) lncRNA in cervical cancer and urothelial cancer associated 1 (UCA1) lncRNA in hypoxia-resistant gastric cancer cells [84,85]. Antisense non-coding RNA in the INK4 locus (ANRIL) is also engaged in tumorigenesis of many tissues. Li et al. (2020) linked the level of miR-7-5p with ANRIL and its role in the progression of T-cell acute lymphoblastic leukemia [86]. LncRNA Cyrano discovered in the brain similarly presents miR-7 sponging capability [87].

### 2.2. MiR-7 Expression and Role in Tissues

MiR-7 is particularly critical in tissue of neuroendocrine origin, such as pancreas or brain. It plays an important role in the development and differentiation of those organs [39]. The hypothalamus and pituitary gland are especially enriched in miR-7, in contrast to the cerebellum, cerebral cortex, striatum, or substantia nigra, where its expression is lower. Such an arrangement may be the result of the presence of *MIR7-3* in the intron of the *PIT1*-encoding gene. In the pituitary gland, miR-7 is involved in regulating the secretion of follicle-stimulating hormone (FSH) and luteinizing hormone (LH) via the prostaglandin F2 receptor negative regulator (PTGRF) [39]. Moreover, miR-7 represses the translation of paired box gene 6 (*PAX6*; important factor in brain and eye organogenesis) by targeting two sites in the 3′UTR [88]. MiR-7 regulates genes involved in repairing neurons [89] and enables synaptic plasticity [90]. Considering the importance of miR-7 in brain tissue, it is not surprising that its downregulation results in the occurrence of pathological conditions like Parkinson’s disease [91] and brain tumors [92,93,94,95]. On the other hand, upregulation of miR-7 is correlated with progression of Alzheimer’s disease [96], schizophrenia [97], and neuroinflammatory processes [98,99].

In the human pancreas, the highest expression of miR-7 occurs between 13 and 18 weeks of gestation. It is correlated with a hormone secretion boost [100]. In the adult pancreas, the islets are characterized by the greatest expression of miR-7 [101], where it regulates proliferation via targeting regenerating islet-derived (Reg) proteins [102]. Moreover, miR-7 is actively engaged in regulation of insulin secretion and its decreased level is correlated with the development of diabetes [103,104]. Lower expression is also observed in pancreatic cancer and might serve as a putative biomarker of disease progression [105,106].

An altered pattern of miR-7 expression and its influence on tumor progression is observed in other types of cancer like breast cancer [51,107], lung cancer [108], melanoma [109], colorectal cancer [110], and hepatocellular carcinoma [111]. Association of miRNA with the formation of multiple tumors may result from a wide range of activity and involvement in primary cellular pathways. MiR-7 regulates proliferation and protects organs against excessive growth. Downregulation of miRNA in tumors leads to uncontrolled proliferation.

Osteosarcoma patients with low levels of miR-7 have poor prognosis [112]. Additionally, cell lines originating from osteosarcoma exhibit enhanced proliferation in comparison to normal osteoblastic cells [112]. Xia et al. (2018) observed that in pancreatic cancer, miRNA-7 causes intensified proliferation through targeting of MAP3K9, hence suppressing NF-κB and MEK/ERK pathways [106]. In pancreatic cancer, increased proliferation is also a consequence of disruption of the EGFR/STAT3 signaling pathway.

Another type of cancer with disruption in the miR-7 status and poor patient outcome is colorectal cancer (CRC). A depletion of miR-7 in CRC also triggers the EGFR pathway [113,114]. Moreover, miR-7 inhibits Krüppel-like factor 4 (KLF4), which acts as an oncogene transcription factor [115]. Another target in CRC is X-ray repair cross complementing 2 (XRCC2), a DNA-repair protein [110]. MiRNA-7 increases proliferation, migration, and angiogenesis through its targets, while decreasing apoptosis in CRC [110,114,115]. In addition, miR-7 blocks metastasis via thyroid receptor interactor protein 6 (TRIP6) [116].

In gastric cancer, miR-7 reduces proliferation and increases apoptosis. However, Lin et al. (2020) linked this function with suppression of Raf-1 [117]. Shi et al. (2014) noticed that expression of both, miR-7 and proteasome activator subunit 3 (REGγ; PSME3), is interfered. REGγ is a direct target for miR-7 and its silencing reduces proliferation and triggers apoptosis [118]. Meanwhile, Wang et al. (2019) investigated the influence of sponging of miR-7 on acceleration of proliferation and migration in triple-negative breast cancer. They associated these findings with serine/threonine-protein kinase PAK 1, which is a direct target for miRNA [70]. Furthermore, miR-7 directly binds to focal adhesion kinase (FAK) triggering downstream effects. In accordance with this, cells showed reduced migration and invasiveness [119]. On the other hand, Yin et al. (2019) found that in glioblastoma, the special AT-rich binding protein 1 (SATB1) is frequently overexpressed. They linked SATB1 with cell migration and invasiveness through its direct impairment by miR-7-5p. The level of miR-7 is also reduced in glioblastoma microvasculature [120]. Restoring its amount prevents extensive proliferation of vascular endothelial cells by targeting Raf-1 [121]. MiR-7 suppresses angiogenesis even in murine xenograft glioblastoma [122]. In melanoma, proliferation and metastasis is inhibited by restoring miR-7 through RELA/NF-κB [109]. In contrast, in non-small cell lung cancer, miR-7 inhibits growth and metastasis via the NOVA alternative splicing regulator 2 (NOVA2) [108] and Bcl-2, a critical regulator of apoptosis [123].

The majority of reports indicate a suppressor role of miR-7 in neoplastic diseases. Restoring the level of miRNA-7 suppresses proliferation and invasiveness and induces apoptosis, reducing malignancy of tumor cells. Since miR-7 is involved in regulation of expression of multiple genes, disrupting its endogenous levels leads to changes in essential signaling pathways. This observation indicates that miR-7 might be a key player in the development of MDR in cancer cells.

## 3. Carcinogenesis and miR-7

**Figure 3:**
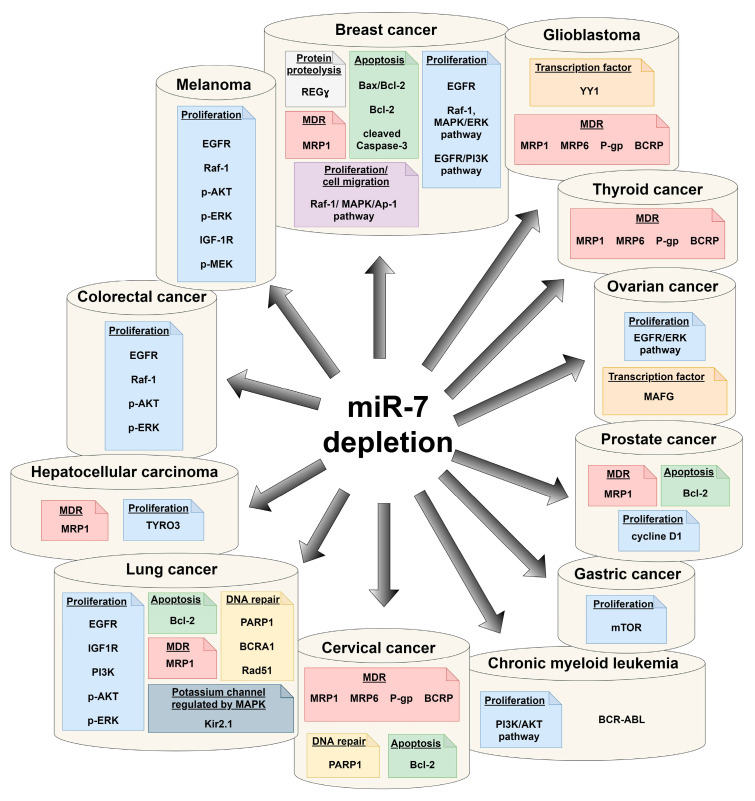
Tumor-related signaling pathways and proteins associated with depletion of miR-7.

It has been observed that in developed countries, lung, sex steroid-responsive breast and prostate tumors, and colon cancer comprise nearly half of newly diagnosed malignancies. Despite progress in therapy strategies, an additional and alarming problem is the phenomenon of multidrug resistance of cancer cells—MDR. It has been shown that expression of miR-7 and carcinogenesis, including modulation of MDR, can be associated (Figure 3).

### 3.1. Breast Cancer

Breast cancer (BC) is the most common type of tumor diagnosed in women [124]. BC falls into at least four subtypes with different molecular backgrounds. The first two subtypes are luminal A and B with high expression of estrogen and/or progesterone receptors (ER and PR, respectively). The third group of BC is the HER2-positive subtype, which shows high expression of HER2 without overexpression of ER and PR. The fourth type, triple-negative breast cancer (TNBC), demonstrates negative results for ER, PR, and HER2 during histopathological characteristic [125]. The prognosis of patients depends on both, subtype and stage of cancer. The five-year survival parameter is high for the I stage, but dramatically drops for the IV stage [124]. Personalized therapies for HER2-positive and ER/PR subtypes are available, whereas there is no personalized treatment for triple-negative BC [126,127]. Around quarter of women with early-stage BC experience recurrent disease and resistance to chemotherapeutic drugs [128,129].

Yang et al. (2019) conducted a study on BC patients who underwent vinorelbine and cisplatin chemotherapy regimen with subsequent mastectomy. The authors found that the level of ciRS-7 (CDR1as; a reported miR-7 regulator) and development of MDR are positively correlated. Moreover, this observation was confirmed using cell lines representing luminal A and triple-negative BC subtypes. It was shown that treatment of these cells with cisplatin in order to achieve resistant cell lines was associated with increase in ciRS-7 and significant reduction in miR-7 expression. Consequently, silencing of ciRS-7 resulted in upregulation of miR-7 and sensitization of cells to the drugs. It was confirmed that augmentation of miR-7 in nude mice with cancer xenografts results in a lower Ki-67 index. The group also managed to prove that REGγ possesses a direct binding site for miR-7, indicating its involvement in the development of drug resistance [130].

Microarray analysis of miRNA expression performed on the MCF-7 and cisplatin-resistant MCF-7 cells revealed significant alterations in the miRNAs’ network associated with the development of MDR. It was established that miR-7 was downregulated in cisplatin-resistant cells and the ABC drug efflux pump MRP1 (*ABCC1*) was identified as a direct target for miR-7 [131]. The link between drug resistance and miR-7 was supported by Gao et al. (2017, 2019). They showed that a reduced level of miR-7 correlates with elevated expression of circ_0006528 in DOX-resistant cell lines and BC tissues. Restoring the miR-7 level via silencing of circ_0006528 or transfection with miR-7 mimic increased the sensitivity of the tested cell lines to the drug [65,66]. Similar research performed by Huang et al. (2019) confirmed Gao’s observations [132]. However, while Gao et al. [66] linked this phenomenon with the effect of silencing of the Raf-1 proto-oncogene (serine/threonine kinase), Huang et al. [132] pointed to alterations in the activity of the EGFR/PI3K signaling pathway.

Uhr et al. (2018) also studied the development of drug resistance in BC and highlighted the importance of alterations in the expression of CDR1-as and miRNA-7. However, they suggested that miR-7 might be associated with poor prognosis, especially in patients with ER-positive tumors. It was found that the level of miR-7 and resistance to tamoxifen in ER-positive patients are negatively correlated [133]. In another trial, Raychaudhuri et al. (2017) performed studies to identify miRNAs involved in response to neo-adjuvant therapy of primary invasive breast cancer. After the treatment regimen, which consisted of epirubicin and paclitaxel (PAX) or epirubicin and cyclophosphamide followed by docetaxel (DTX), all patients had surgical resection followed by adjuvant therapy with cyclophosphamide, fluorouracil, and methotrexate. Hormone receptor-positive patients received tamoxifen over a period of 5 years. Patients with a history of lumpectomy underwent radiotherapy. All specimens underwent histopathological examination to assess patients’ response to therapy. The performed quantitative real-time PCR allowed for selection of miR-7 and miR-340 as candidates for evaluation of response to treatment. The reported data suggest that lower expression of miR-7 and higher expression of miR-340 result in a positive outcome of neo-adjuvant therapy [134].

Hong et al. (2019) observed that patients with higher levels of miR-7 manifest better response to chemotherapy. The combination of paclitaxel and carboplatin is a neo-adjuvant regimen commonly used for patients with metastatic breast cancer. The evaluated response to treatment with both drugs prior to surgical resection resulted in pathological complete response (pCR) in one subgroup of patients, while some patients did not respond to therapy. Further analysis revealed that pCR patients manifested higher levels of miR-7 expression. It was observed that miR-7 expression was significantly lowered in the established paclitaxel-resistant BC-derived (MCF-7) cells, which indicates that miR-7 can sensitize cells to drugs. Transcripts of *ABCC1* (MRP1) and *BCL-2* (Bcl-2, apoptosis regulator) were reported to be targets of miR-7 [135].

Expression of HER2, especially the HER2Δ16 isoform, is another unfavorable factor correlated with poor prognosis in BC. The HER2Δ16 isoform remains constantly active and is present in about 50% of HER2-positive patients. Huynh and Jones (2014) conducted an analysis on miRNA expression in the MCF-7 cell line transfected with HER2Δ16. The performed miRNA microarray analysis revealed that miR-7 was significantly downregulated in HER2Δ16-overexpressing MCF-7 cells. Moreover, HER2Δ16-transfected cells demonstrated an increased expression of EGFR. Restoring the level of miR-7 resulted in a reduction of colony formation and decelerated migration of cells. This was caused by interference in multiple signaling pathways that involve EGFR and Src kinase activity. Since the HER2Δ16 isoform prompts trastuzumab resistance, the group verified the role of miR-7 in MDR. It was found that restoring the miR-7 level in the HER2Δ16-overexpressing cells reversed trastuzumab resistance and significantly sensitized cells to the drug [136].

Lapatinib is a tyrosine kinase inhibitor (TKI) that has dual action and suppresses both EGFR and HER2 receptors. The most promising clinical outcomes are achieved in HER2-positive patients, while TNBC patients have worse outcomes after therapy. Since overexpression of EGFR is connected with suppression of miR-7, Hsiao et al. (2015) investigated whether disrupted levels of miRNA might be related to failure of lapatinib therapy in TNBC. The authors concluded that downregulation of miR-7 affects the Raf-1/MAPK/Ap-1 pathway and in consequence, impacts the expression of interleukin-6 (IL-6). Augmentation of miR-7 sensitized both, the BC-derived MDA-MB-231 and lapatinib-resistant MDA-MB-231 cells to the drug. It was proposed that up-regulated miR-7 is capable of impairing lapatinib-mediated production of IL-6 through direct binding to Raf-1 [137].

### 3.2. Lung Cancer

Lung cancer (LC) is the major cause of cancer-related death in both women and men. Two main groups are defined: non-small cell lung cancer and small cell lung cancer (SCLC). NSCLC is diagnosed in approximately 85% of all LC patients. NSCLC exhibits a similar pattern of behavior and response to therapy as adenocarcinoma, large cell carcinoma, and squamous cell carcinoma [138,139]. In contrast, SCLC is significantly less frequently diagnosed and is classified as a neuroendocrine tumor [140].

Treatment of LC is a serious therapeutic challenge, mainly due to the frequently observed high resistance of cells to chemotherapeutic agents. Ge et al. (2015) investigated the profile of miRNAs in LC-derived gefitinib-sensitive and gefitinib-resistant cells. The microarray data showed altered expression of several microRNAs, including miR-7, in gefitinib-resistant cells. Since gefitinib is a direct inhibitor of EGFR, it was proposed that miR-7-mediated altered expression of EGFR may trigger the development of the resistance phenotype. As expected, transfection of the resistant cell line with miR-7 mimic reduced the expression of the EGFR protein to the level reported in the drug-sensitive cells [141].

Other groups also studied the role of miR-7 in NSCLC. Zhao et al. (2015) confirmed the role of miR-7 and EGFR in overcoming gefitinib sensitivity. They showed that co-treatment of NSCLC-derived cells with miR-7 mimic and the drug resulted in more efficient inhibition of cell growth caused by alterations in components of the EGFR pathway. Lower expression of Raf-1 and decreased phosphorylation of ERK and AKT were observed. Moreover, cells transfected with miR-7 exhibited G0/G1 phase arrest and increased apoptosis. The authors concluded that miR-7 increases cytotoxicity of the drug [142]. Mou et al. (2016) examined the level of miR-7 in sera of NSCLC patients and assumed that the miR-7 level is correlated with the stage of the disease and response to therapy. The study included two groups: (i) patients with the I/II stages who underwent surgery and gefitinib treatment and (ii) patients with the III/IV stages treated only with gefitinib. The tested level of miRNAs (in both tissue samples and sera) showed that patients with the I/II stages presented lower levels of miR-7 in comparison to patients with the III/IV phases of the disease. The authors observed a decrease in miR-7 expression during therapy in both groups, however, the difference in the level of miR-7 before and after treatment among patients with the I/II stages was lower than the difference reported in patients with the III/IV tumor stage. Moreover, progression-free survival was better for patients with low levels of miR-7 [143].

In contrast, Cheng et al. (2017) concluded that lower expression of miR-7 could be a prognostic factor for poor survival. The performed studies revealed that the level of miR-7 remained decreased in the tested LC specimens. Analysis showed that patients with higher expression of miR-7 presented a better 5-year survival rate. A test performed on the lung adenocarcinoma-derived SPC-A1 cell line showed that presence of miR-7 caused slower proliferation and enhanced apoptosis, especially when cells were co-treated with cisplatin. The authors also silenced Bcl-2 and observed induction of cisplatin-induced apoptosis. In contrast, cells transfected with miR-7 mimic and harboring the Bcl-2 expression vector were more sensitive to the drug. It was confirmed that miR-7 directly binds to 3′UTR of Bcl-2. The authors proposed that cisplatin-induced apoptosis in SPC-A1 resistant cells might be caused by inactivation of Bcl-2 by miR-7 [144].

Another group, He et al. (2015), performed a study on the effect of docetaxel on NSCLC-derived cell lines. Cells treated with DTX presented elevated expression of miR-7. A similar effect was not observed for the other miRNAs. The group suggested that usage of inhibitor(s) of miR-7 might increase the effectiveness of chemotherapeutic-based treatment procedures [145]. In a similar study, Liu et al. (2014) investigated the link between NSCLC-derived cells’ sensitivity to PAX and endogenous expression levels of miR-7. It was shown that higher abundance of miR-7 correlates with a lower drug dose necessary to induce apoptosis. Consequently, cells transfected with miR-7 mimic presented reconstituted sensitivity to PAX due to downregulation of EGFR [146].

Patients suffering from SCLC initially respond quite well to the therapy, but the 2-year survival rate remains below 5%, mainly due to the development of resistance [147]. Multidrug-resistant lung cancer cell line H69AR presents lower expression of miR-7 in comparison to the sensitive H69 cells [148]. In addition, studies on tissue samples confirmed that patients sensitive to treatment have a higher expression of miR-7 compared to patients resistant to therapy. Expression of miR-7 was correlated with MRP1 activity, as a high level of the MRP1 protein was observed in samples with depletion of miR-7. Liu et al. (2015) investigated the role of miRNAs in triggering drug resistance. They observed direct binding of miR-7 to the target site of the 3′UTR sequence of the *ABCC1* gene. Additionally, transfection of the multidrug-resistant H69AR cells with miR-7 mimic resulted in lower expression of the *ABCC1* (MRP1) gene. On the other hand, transfection of drug-sensitive H69 cells with miR-7 inhibitor enhanced *ABCC1* expression. These data confirmed the significant role of miR-7 in regulation of the drug efflux transporter MRP1 and managing MDR [147].

Immunohistochemical analysis performed by Liu et al. (2015) showed correlation between presence of Kir2.1, the progression of the disease and drug resistance. They revealed that patients with higher expression of Kir2.1 were less responsive to therapy. Kir2.1 is a member of the potassium channel family encoded by the *KCNJ2* gene and can affect key cellular processes including proliferation, adhesion or apoptosis. Since MRP1 is engaged in drug resistance in SCLC, the authors also confirmed (through immunoprecipitation) that Kir2.1 interacts with the MRP1 protein. It was proved that miR-7 not only binds directly to the 3′UTR sequence of *ABCC1*, but can also regulate Kir2.1 by targeting the 3′UTR of the *KCNJ2* gene. It was concluded that negative correlation between miR-7 and Kir2.1 levels might be considered as a predictive factor and linked with patients’ positive prognosis [149].

Lai et al. (2019) showed that resistant SCLC cells transfected with miR-7 present higher sensitivity to doxorubicin. DOX is one of the most common standard chemotherapeutics, which damages the cell’s DNA via induction of double-strand breaks. As homologous recombination (HR) is the only way of repairing the damaged genome, the role of the *PARP1* gene product was investigated. *PARP1* encodes for the poly (ADP-ribose) polymerase 1 (PARP1) protein, which is a key player involved in the HR process. Researchers predicted putative binding sites for miR-7 in the 3′UTR of PARP1 mRNA. Moreover, it was shown that drug-resistant cells transfected with miR-7 mimic present reduced expression of the *PARP1* gene, whilst miR-7 inhibition triggers PARP1 expression. Therefore, it can be considered that pre-targeting tumors presenting high expression of PARP1 with miR-7 may increase the effectiveness of the administered chemotherapeutics [150].

### 3.3. Glioblastoma

Glioblastoma is an extremely invasive and malignant primary brain tumor. Despite the availability of therapeutic approaches, the outcome remains poor and patients’ survival and prognosis have not improved over the years. The median life-time after diagnosis is approximately 10 months and the 5-year survival is still at 5.1% [151]. The therapeutic options for the treatment of glioblastoma are extremely limited. A significant challenge in GB treatment is the presence of the blood brain barrier (BBB). The BBB is highly selective, thus only small, lipophilic molecules are able to enter the central nervous system (CNS). The first-line drugs used in glioblastoma therapy are temozolomide (TMZ) and carmustine (BCNU). Nevertheless, the survival of patients treated with TMZ post-surgery is roughly 2 months longer [152]. In addition, fast development of MDR by GB cells is another important issue negatively affecting the outcome of treatment.

As already mentioned, miR-7 plays a crucial role in the development of the CNS and additionally, its important role in GB progress was also highlighted. Various groups reported significantly decreased levels of miR-7 in GB tissues [92,153]. Jia et al. (2019) linked miR-7 and GB resistance to TMZ. They observed a significant reduction in the miR-7 level in the TMZ-resistant cell line. Augmentation of miR-7 resulted in decreased spheroid formation. Moreover, miR-7 impaired the self-renewal of TMZ-resistant glioblastoma stem cells, which also affected tumor proliferation. The group confirmed that miR-7 directly binds to the 3′UTR sequence of the Yin Yang 1 (YY1) transcription factor. TMZ-resistant cells with knocked-down *YY1* gene exhibited slower growth and colony formation. The in vivo study showed that administration of miR-7 and TMZ causes reduction in tumor size in mice compared to TMZ administered alone or no treatment [153].

Our own studies also highlighted the role of miR-7 in the development of drug resistance in GB [92]. The experiments were performed using GB-derived drug-sensitive cells (A172), as well as the T98G drug-resistant cell line. It was found that miR-7 sensitizes both cell lines to various drugs. A significant reduction in cell viability was observed in cell lines treated simultaneously with miR-7 mimic and drug (DOX or TMZ or BCNU). The therapeutic effect of combined treatment of cells with miRNA and drug was even more spectacular when cells were exposed to TMZ/miR-7-loaded nanoparticles (cubosomes). It was also confirmed that the observed effect of miR-7/drug co-treatment is not cell line-specific. The outcome of treatment of the thyroid carcinoma cell line TPC-1 and HeLa cells was similar to that observed for GB-derived cells. The effect of miR-7 in overcoming drug resistance was linked with possible alterations in the expression of efflux pumps. It was confirmed that expression of *ABCC1*, *ABCC6*, *ABCB1*, and *ABCG2* (encoding for MRP1, MRP6, P-gp, and BCRP, respectively) was significantly enhanced in patients’ specimens. Moreover, it was shown that treatment of drug-resistant T98G cells with miR-7 mimic results in downregulation of key multidrug efflux pumps. It was concluded that the effectiveness of combinatory treatment with miR-7 and chemotherapeutics likely results from miR-7-mediated suppression of MDR-encoding genes [92].

### 3.4. Hepatocellular Carcinoma

Hepatocellular carcinoma is the most malignant liver cancer [154]. The incidence is on the rise and the only option currently available for HCC patients is treatment with sorafenib, a multikinase inhibitor. Kabir et al. (2018) investigated the development of resistance to sorafenib using the HCC-originated sorafenib-sensitive and sorafenib-resistant cell lines. The obtained data showed that in inhibitor-treated cells, changes in PI3K/AKT and Raf-1/MEK/ERK signaling pathways occurred. The observed effects on components of signaling pathways were accompanied by depletion of miR-7, for which the role of a tumor suppressor was proposed. Its expression was significantly reduced in sorafenib-resistant cells, which also presented impaired migration and proliferation. The *TYRO3* gene was identified as a binding target for miR-7. This member of the transmembrane tyrosine kinase receptors is upregulated in HCC patients. The tests performed on HCC-derived cell lines also confirmed TYRO3 upregulation, especially in sorafenib-resistant cells. It was confirmed that depletion of TYRO3 expression in sorafenib-resistant cells combined with administration of the drug, significantly increases the sensitivity of cells to the chemotherapeutic agent. A similar effect was obtained during treatment of sorafenib-sensitive cells with miR-7 mimic and inhibitor [155]. Another study conducted by Hu et al. (2018) also confirmed the role of miR-7 in overcoming oxalipatin resistance in HCC cells. The miR-7/MRP1 interaction was proposed as a molecular mechanism involved in the development of the MDR phenotype [78].

### 3.5. Ovarian Cancer

Ovarian cancer (OC) is one of main causes of cancer-related death among women, mainly due to late diagnosis. Another unfavorable factor limiting successful therapy, and therefore contributing to the high mortality rate, is the high genetic and molecular heterogeneity of OC [156]. Paclitaxel is an apoptosis-inducing drug commonly used in the treatment of ovarian cancer. However, the disadvantage of PAX is induction of the ERK pathway, which frequently leads to the development of drug resistance. Cui et al. (2018) performed studies on the role of miR-7 in overcoming PAX resistance using paclitaxel- and miR-7-loaded nanoparticles. The obtained data indicated a significant increase in sensitivity of OC cells to the chemotherapeutic in the presence of miR-7 [157].

Cisplatin is a widely used drug for treatment of various cancers, which also triggers the development of MDR. Epigenetic changes, including disruption of miRNAs’ network, are considered as one of the possible mechanisms responsible for MDR. Vera et al. (2017) performed a study on various OC-derived cells and patients’ specimens to evaluate the correlation of miRNAs’ expression and development of resistance to cisplatin. The examination of microarray data enabled the selection of miR-7 as one of the most significantly suppressed targets in cisplatin-resistant cells. It was also observed that in drug-resistant A2780R cells, the aberrant methylation of the *MIR-7* promoter was greater than in the drug-sensitive A2780S cell line. Additionally, the miR-7 methylation status in OC tissue samples was enhanced. The transcription factor MAFG was identified as a direct target of miR-7. It was shown that overexpression of the *MAFG* gene resulted in an increased resistance to the drug in cisplatin-sensitive A2780S cells [158].

### 3.6. MiR-7 in Other Cancers

Similar to the previously discussed cancer types, enhancement of miR-7 expression was linked with limited effectiveness of various therapies (Table 2) including cisplatin treatment of gastric cancer (GC). Xu et al. (2017) confirmed that in GC-derived cell lines and tissue samples obtained from patients, expression of miR-7 was downregulated. In addition, the level of miR-7 was lowered in cisplatin-resistant GC specimens. Augmentation of GC cells with miR-7 mimic impaired both proliferation and invasion. Bioinformatics analyses allowed for selection of mTOR, serine/threonine kinase, as a potential target of miR-7. In addition, it was found that expression of mTOR is suppressed in cells transfected with miR-7 mimic. The performed viability test and flow cytometry analysis showed increased sensitivity of miR-7 pre-treated cells to cisplatin [159].

Yang et al. (2018) came to opposite conclusions concerning the role of miR-7 in cervical cancer (CC). Cisplatin is also the first-choice chemotherapeutic in the treatment of advanced stages of CC, however, acquired resistance limits the successful outcome of treatment. In their studies, Yang et al. noticed that in the majority of analyzed patients’ samples, the expression of miR-7 was significantly suppressed. However, in in vitro experiments using cisplatin-resistant HeLa cells, miR-7 expression was enhanced in comparison to the levels observed in the parental cisplatin-sensitive HeLa. Further, silencing endogenous miR-7 enhanced apoptosis after treatment of cells with cisplatin. The authors indicated that suppression of miR-7 results in an increased level of DNA repair marker γH2AX and a reduced ATP/ADP ratio in cisplatin-resistant cells. Moreover, they noticed that miR-7 downregulates PARP1 expression, and therefore, inhibition of miR-7 may promote PARP1-mediated DNA repair. It was confirmed that silencing PARP1 expression results in a greater apoptosis rate after administration of cisplatin. The researchers also noticed, that miR-7 increases autophagy and that suppression of both PARP-1 and Bcl-2 prompts the process of autophagy [160]. These data stay in contradiction with reports published by Lai et al. [150] and Hong et al. [135] with regards to the role of PARP1 and Bcl-2 in drug resistance.

Prostate cancer (PC) is one of the most common causes of cancer-related death in men. Treatment strategies for advanced stages of the disease include androgen deprivation. Unfortunately, most PCs can develop resistance to the treatment regimen, which is known as castration-resistant prostate cancer (CRPC). In such patients, docetaxel is a first-line treatment. Gao et al. (2020) investigated the effect of circular RNA hsa_circ_0000735 on prostate cancer. They observed that expression of hsa_circ_0000735 was higher in PC patients’ samples and it was correlated with poor prognosis. The researchers also studied PC-originated cells (including DTX-resistant lines) and found that expression of hsa_circ_0000735 was elevated in drug-resistant cells and thus, silencing this circRNA re-sensitized cells to DTX. Gao et al. also reported that hsa_circ_0000735 acts as a sponge for miR-7 in DTX-resistant cells. Downregulation of hsa_circ_0000735 boosted the miRNA level and made resistant cell lines more prone to the drug treatment by decreasing cell viability and colony formation. In addition, reducing miR-7 expression, which was mediated by the studied circRNA knockdown, partially deprived cells of their sensitivity to the drug. The scientists managed to link miR-7 activity with reduced levels of P-gp, Bcl-2, and cycline D1. The results were also confirmed in vivo using the nude mouse xenograft model [68].

Chronic myeloid leukemia (CML) is a malignant disease of hematopoietic stem cells. The most common aberration in this disease is the Philadelphia chromosome (t(9;22)(q34;q11) translocation), which results in the *BCR-ABL* fusion. The standard agents employed for CML are tyrosine kinase inhibitors that suppress signaling pathways triggered by *BCR-ABL*. Imatinib is an inhibitor commonly used as a first-line therapy. However, treatment strategies of CML are limited due to the development of the MDR phenomenon. MiR-7 is known to be a suppressor of the PI3K/AKT pathway, which is disrupted in CML. Jiang et al. (2017) investigated the properties of miR-7 in imatinib-treated cells. The researchers showed that the expression of miR-7 was significantly upregulated in CML-derived K562 cells in contrast to the expression observed in white blood cells obtained from healthy donors. In addition, cells transfected with the inhibitor were resistant to the treatment. In the miR-7-transfected cell line, the BCR-ABL protein was downregulated and BCR-ABL was marked as a target for miR-7, with a binding site within the 3′UTR sequence of *ABL* [161].

Colorectal cancer and melanoma are other cancers with miR-7-mediated disruption of EGFR and PI3K/AKT pathways. In melanoma, miR-7 restores sensitivity to BRAF inhibitors (BRAFi), such as vemurafenib and dabrafenib. Mutations of *BRAF* are widely observed in metastatic melanomas. Treatment with BRAFi initially gives promising results, however the relapse ratio remains high. Sun et al. (2016) revealed that the established vemurafenib-resistant cell lines exhibited lower expression of miR-7 compared to the parental cell lines. Additionally, transfection with the miR-7 mimic oligonucleotide sensitized resistant cell lines to therapy with inhibitors and impaired cell viability. MiR-7 was also shown to suppress the progression of vemurafenib-resistant melanoma in mice [162]. In contrast, in colorectal cancer, expression of miR-7 was higher in tumor samples than in adjacent tissues without signs of malignancy. However, overall survival of patients was worse among patients with low expression of miR-7 in tumor samples and much better in patients with high expression of miR-7. Additionally, when Suto et al. (2015) transfected colorectal cancer-derived HTC116, SE480, and HT29 cell lines with the miR-7 precursor and miR-7 inhibitor, they found that the proliferation rate dropped in the miR-7-overexpressing cell lines with the *KRAS* mutation. Concurrently, co-treatment of *KRAS*-mutated cells (naturally resistant to cetuximab) with pre-miR-7 and cetuximab, an antibody targeting the EGFR protein, resulted in sensitizing the cells to antibody therapy and slower cell growth. The *BRAF*-mutated HT29 cell line treated with the miR-7 precursor exhibited no change in sensitivity to the drug. Suto et al. also proved that miR-7 targets EGFR and Raf-1 [113]. Both groups confirmed that miR-7 lowers EGFR, Raf-1, phospho-AKT, and phospho-ERK proteins and impairs downstream pathways [113,162].

Ye et al. (2020) discovered that miR-7 might be a predictive biomarker of gemcitabine therapy in pancreatic ductal adenocarcinoma (PDAC). They divided patients into gemcitabine-sensitive and gemcitabine-resistant groups and analyzed serum samples. Gemcitabine-sensitive patients had greater expression of miR-7 than patients from the other group. Based on analysis of The Cancer Genome Atlas (TCGA), low expression of miR-7 was observed in patients with an advanced stage of PDAC and correlated with a worse prognosis. Tests performed on PDAC-derived cell lines transfected with miR-7 mimic confirmed a better response to gemcitabine when miRNA was overexpressed. Cells with exogenous miRNA were prone to formation of colonies and their viability was reduced [105]. 

## 4. Conclusions and Future Perspective

Over the past decades, a significant progress in the study of miRNAs and their role in cell physiology and pathogenesis has been highlighted. MiRNA-7 deserves special attention, as its sequence is strongly conserved between species. MiRNA-7 is downregulated in most cancer types and its depletion affects the expression of various proteins involved in apoptosis, EGFR signaling pathways, and MDR. In most cases, miRNA-7 has been shown to increase the sensitivity of tumor cells to the therapeutic agents [92,135,146]. Therefore, miR-7 can be considered as a very promising putative target in cancer therapy.

According to the PATENTSCOPE database (patentscope.wipo.int), there are thousands of miRNA-related patents, including 31 related to miR-7. MiRNAs have a wide range of applications, from diagnostic tests and biomarkers to potential tools for treatment. Still, the major obstacle in implementation of miRNA-based therapy is efficient and targeted delivery of miRs to the pathological lesions. Naked miRNA is easily degraded by endogenous nucleases and poorly passes through the cell membrane due to its negatively charged structure. Therefore, research is focused on increasing the stability of miRNAs and creating vehicles (e.g., liposomes, polymers, exosomes, or viral carriers) capable of efficient delivery of miRNAs [163]. To increase effectiveness of miR-loaded transporters, further modifications of their surface properties, like presence of thiol-reactive groups, lipid tails, specific ligands, or antibodies (binding to receptors), are proposed [164,165,166,167].

Currently, there are ongoing clinical trials on miRNA-based therapies that have reached phase I or II. Both miRNA mimics and inhibitors (antimiRs) are examined [168]. The studies on RG012, an antimiR of miR-21, in treatment of Alport syndrome, recently reached the II phase (ClinicalTrials.gov Identifier: NCT02855268). Another drug, Miravirsen (antimiR of miR-122), designed for treatment of chronic hepatitis C, also gave promising results (ClinicalTrials.gov Identifier: NCT01200420) [169]. Remlarsen (MRG-201, ClinicalTrials.gov Identifier: NCT03601052) is a promising miR-29a mimic drug for treatment of cutaneous fibrosis. Additionally, in this review, we described two studies concerning co-delivery of miR-7 and a drug loaded in nanovehicles, which sensitized cancer cells to the chemotherapeutic [92,157]. However, both nanocarriers must undergo further investigations.

Overall, miRNAs, including miR-7, are promising therapeutic targets. However, the future of successful engagement of miRNAs in therapy depends on the effectiveness of delivery systems.

## Figures and Tables

**Table 1:** The list of reported miR-7 regulators in cancer.

I. Transcriptional Level				
Transcription Factors	*MIR7-1*	*MIR7-2*	*MIR7-3*	References
**c-Myc**	↑	—	—	[46]
**HOXD10**	↑	—	—	[47]
**HNF4** **α**	—	↑	—	[48]
**FOXP3**	↑	↑	—	[49]
**RELA**	↓	↓	—	[50,51,52]
**Usp18**	↓	↓	↓	[53]
**II. Post-Transcriptional Level**				
**pri-miRNA**				
**Factor**	**pri-miR-7-1**	**pri-miR-7-2**		
**HuR/MSI2**	↑	—	—	[54]
**QKI-5**	↓	—	—	[55]
**QKI-6**	↓	—	—	[55]
**SF2/ASF**	↑	—	—	[56]
**Mature miRNA Level**				
**ceRNA**	**Regulation**	**Confirmed in:**		
**circRNA**				
**ciRS-7**	↓	brain, non-small cell lung cancer, esophageal squamous cell carcinoma, papillary thyroid cancer, colorectal cancer		[59,60,61,62,63]
**circSNCA**	↓	brain		[64]
**circ_0006528**	↓	breast cancer		[65,66]
**circ-U2AF1**	↓	glioma		[67]
**circ_0000735**	↓	prostate cancer		[68]
***circ-ITCH***	↓	osteosarcoma		[69]
**circ-TFCP2L1**	↓	breast cancer		[70]
**circ_0015756**	↓	hepatocellular carcinoma		[71]
**lncRNA**				
**CASC21**	↓	colorectal cancer		[73]
**RHPN1-AS1**	↓	colorectal cancer, hepatocellular carcinoma		[74,77]
**RP4**	↓	colorectal cancer		[75]
**TINCR**	↓	colorectal cancer		[76]
**KCNQ1OT1**	↓	hepatocellular carcinoma		[78]
**LINC00115**	↓	triple-negative breast cancer, lung adenocarcinoma		[79,80]
**MEG3**	↓	renal cell carcinoma		[81]
**HOTAIR**	↓	breast cancer		[82]
**FOXD2-AS1**	↓	thyroid cancer		[83]
**SOX21** **-** **AS1**	↓	cervical cancer		[84]
**UCA1**	↓	gastric cancer		[85]
**ANRIL**	↓	acute lymphoblastic leukemia		[86]
**Cyrano**	↓	brain		[87]

↑, upregulation; ↓, downregulation.

**Table 2:** MiRNA-7 in cancer treatment.

Tumor Type	MiR-7 Level	Drug	Role	Protein	Reference
**Breast cancer**	down	cisplatin	sensitize	REGγ, Bax/Bcl-2, cleaved Caspase-3	[130]
	down	cisplatin	sensitize	MRP1	[131]
	down	doxorubicin	sensitize	Raf-1, MAPK/ERK pathway	[65,66]
	down	doxorubicin	sensitize	EGFR/PI3K pathway	[132]
	up	tamoxifen	resistance	nd	[133]
	up	n.eo-adjuvant therapy: epirubicin, paclitaxel, cyclophosphamide, docextaxel; post-surgery: cyclophosphamide, fluorouracil, methotrexate	resistance	nd	[134]
	down	paclitaxel, carboplatin	sensitize	MRP1, Bcl-2	[135]
	down	trastuzumab	sensitize	EGFR	[136]
	down	lapatinib	sensitize	Raf-1/MAPK/Ap-1 pathway	[137]
**Lung cancer**	down	gefitinib	sensitize	EGFR	[141]
	down	gefitinib	sensitize	IGF-1R, PI3K, p-AKT, p-ERK	[142]
	up	gefitinib	resistance	nd	[143]
	down	cisplatin	sensitize	Bcl-2	[144]
	down	docetaxel	sensitize	nd	[145]
	down	paclitaxel	sensitize	EGFR	[146]
	down	nd	sensitize	MRP1	[147]
	down	cisplatin, doxorubicin, etoposide	sensitize	Kir2.1	[149]
	down	doxorubicin	sensitize	PARP1, BCRA1, Rad51	[150]
**Glioblastoma**	down	temozolomid	sensitize	YY1	[153]
	down	temozolomid, doxorubicin, carmustine	sensitize	MRP1, MRP6, P-gp, BCRP	[92]
**Thyroid cancer**	down	temozolomid, doxorubicin	sensitize	MRP1, MRP6, P-gp, BCRP	[92]
**Cervical cancer**	down	temozolomid, doxorubicin	sensitize	MRP1, MRP6, P-gp, BCRP	[92]
	down/up	cisplatin	resistance	PARP1, Bcl-2	[160]
**Hepatocellular carcinoma**	down	sorafenib	sensitize	TYRO3	[155]
	down	oxalipatin	sensitize	MRP1	[78]
**Ovarian cancer**	down	paclitaxel	sensitize	EGFR/ERK pathway	[157]
	down	cisplatin	sensitize	MAFG	[158]
**Gastric cancer**	down	cisplatin	sensitize	mTOR	[160]
**Prostate cancer**	down	docetaxel	sensitize	P-gp, Bcl-2, cycline D1	[68]
**Chronic myeloid leukemia**	up	imatinib	sensitize	BCR-ABL, PI3K/AKT pathway	[161]
**Colorectal cancer**	down	cetuximab	sensitize	EGFR, Raf-1, p-AKT, p-ERK	[113]
**Melanoma**	down	vemurafenib	sensitize	EGFR, Raf-1, p-AKT, p-ERK, IGF-1R, p-MEK	[162]
**Pancreatic ductal adenocarcinoma**	down	gemcitabine	sensitize	nd	[105]

nd, not defined.

## Data Availability

The data presented in this study are available on request from the corresponding author.

## References

[1] Lee R.C., Feinbaum R.L., Ambros V. (1993). The *C. elegans* heterochronic gene *lin-4* encodes small RNAs with antisense complementarity to *lin-14*. Cell.

[2] Wightman B., Ha I., Ruvkun G. (1993). Posttranscriptional regulation of the heterochronic gene *lin-14* by *lin-4* mediates temporal pattern formation in *C. elegans*. Cell.

[3] Alles J., Fehlmann T., Fischer U., Backes C., Galata V., Minet M., Hart M., Abu-Halima M., Grässer F.A., Lenhof H.-P. (2019). An estimate of the total number of true human miRNAs. Nucleic Acids Res..

[4] O’Brien J., Hayder H., Zayed Y., Peng C. (2018). Overview of MicroRNA biogenesis, mechanisms of actions, and circulation. Front. Endocrinol..

[5] Treiber T., Treiber N., Meister G. (2019). Regulation of microRNA biogenesis and its crosstalk with other cellular pathways. Nat. Rev. Mol. Cell Biol..

[6] Macfarlane L.-A., Murphy P.R. (2010). MicroRNA: Biogenesis, function and role in cancer. Curr. Genom..

[7] Stavast C.J., Erkeland S.J. (2019). The non-canonical aspects of MicroRNAs: Many roads to gene regulation. Cells.

[8] Catalanotto C., Cogoni C., Zardo G. (2016). MicroRNA in control of gene expression: An overview of nuclear functions. Int. J. Mol. Sci..

[9] Russo F., Fiscon G., Conte F., Rizzo M., Paci P., Pellegrini M. (2018). Interplay between long noncoding RNAs and MicroRNAs in cancer. Methods in Molecular Biology.

[10] Condrat C.E., Thompson D.C., Barbu M.G., Bugnar O.L., Boboc A., Cretoiu D., Suciu N., Cretoiu S.M., Voinea S.C. (2020). miRNAs as biomarkers in disease: Latest findings regarding their role in diagnosis and prognosis. Cells.

[11] Anderson C., Catoe H., Werner R. (2006). MIR-206 regulates connexin43 expression during skeletal muscle development. Nucleic Acids Res..

[12] Coffre M., Koralov S.B. (2017). miRNAs in B cell development and lymphomagenesis. Trends Mol. Med..

[13] Chakraborty M., Hu S., Visness E., Del Giudice M., De Martino A., Bosia C., Sharp P.A., Garg S. (2020). MicroRNAs organize intrinsic variation into stem cell states. Proc. Natl. Acad. Sci. USA.

[14] Foshay K.M., Gallicano G.I. (2009). miR-17 family miRNAs are expressed during early mammalian development and regulate stem cell differentiation. Dev. Biol..

[15] Liu Q., Fu H., Sun F., Zhang H., Tie Y., Zhu J., Xing R., Sun Z., Zheng X. (2008). miR-16 family induces cell cycle arrest by regulating multiple cell cycle genes. Nucleic Acids Res..

[16] Ivanovska I., Ball A.S., Diaz R.L., Magnus J.F., Kibukawa M., Schelter J.M., Kobayashi S.V., Lim L., Burchard J., Jackson A.L. (2008). MicroRNAs in the miR-106b family regulate p21/CDKN1A and promote cell cycle progression. Mol. Cell. Biol..

[17] Borden A., Kurian J., Nickoloff E., Yang Y., Troupes C.D., Ibetti J., Lucchese A.M., Gao E., Mohsin S., Koch W.J. (2019). Transient introduction of miR-294 in the heart promotes cardiomyocyte cell cycle reentry after injury. Circ. Res..

[18] Wu J., Qian J., Li C., Kwok L., Cheng F., Liu P., Perdomo C., Kotton D., Vaziri C., Anderlind C. (2010). miR-129 regulates cell proliferation by downregulating Cdk6 expression. Cell Cycle.

[19] Liu C., Teng Z.-Q., Santistevan N.J., Szulwach K.E., Guo W., Jin P., Zhao X. (2010). Epigenetic regulation of miR-184 by MBD1 governs neural stem cell proliferation and differentiation. Cell Stem Cell.

[20] Lal A., Navarro F., Maher C.A., Maliszewski L.E., Yan N., Oday E.M., Chowdhury D., Dykxhoorn D.M., Tsai P., Hofmann O. (2009). miR-24 inhibits cell proliferation by targeting E2F2, MYC, and other cell-cycle genes via binding to “seedless” 3′UTR MicroRNA recognition elements. Mol. Cell.

[21] Singh A.P., Hung Y.-H., Shanahan M.T., Kanke M., Bonfini A., Dame M.K., Biraud M., Peck B.C., Oyesola O.O., Freund J.M. (2020). Enteroendocrine progenitor cell–enriched miR-7 regulates intestinal epithelial proliferation in an *Xiap*-dependent manner. Cell. Mol. Gastroenterol. Hepatol..

[22] Zhang Y., Liu D., Chen X., Li J., Li L., Bian Z., Sun F., Lu J., Yin Y., Cai X. (2010). Secreted monocytic miR-150 enhances targeted endothelial cell migration. Mol. Cell.

[23] Zhuang G., Wu X., Jiang Z., Kasman I., Yao J., Guan Y., Oeh J., Modrusan Z., Bais C., Sampath D. (2012). Tumour-secreted miR-9 promotes endothelial cell migration and angiogenesis by activating the JAK-STAT pathway. EMBO J..

[24] Xu C., Lu Y., Pan Z., Chu W., Luo X., Lin H., Xiao J., Shan H., Wang Z., Yang B. (2007). The muscle-specific microRNAs miR-1 and miR-133 produce opposing effects on apoptosis by targeting HSP60, HSP70 and caspase-9 in cardiomyocytes. J. Cell Sci..

[25] Buscaglia L.E.B., Li Y. (2011). Apoptosis and the target genes of microRNA-21. Chin. J. Cancer.

[26] Lukasik A., Zielenkiewicz P. (2016). Plant MicroRNAs—Novel players in natural medicine?. Int. J. Mol. Sci..

[27] Oliveto S., Mancino M., Manfrini N., Biffo S. (2017). Role of microRNAs in translation regulation and cancer. World J. Biol. Chem..

[28] Tsuruo T., Naito M., Tomida A., Fujita N., Mashima T., Sakamoto H., Haga N. (2003). Molecular targeting therapy of cancer: Drug resistance, apoptosis and survival signal. Cancer Sci..

[29] Housman G., Byler S., Heerboth S., Lapinska K., Longacre M., Snyder N., Sarkar S. (2014). Drug resistance in cancer: An overview. Cancers.

[30] Sun Y.-L., Patel A., Kumar P., Chen Z.-S. (2012). Role of ABC transporters in cancer chemotherapy. Chin. J. Cancer.

[31] Wu S., Fu L. (2018). Tyrosine kinase inhibitors enhanced the efficacy of conventional chemotherapeutic agent in multidrug resistant cancer cells. Mol. Cancer.

[32] Longley D.B., Johnston P.G. (2005). Molecular mechanisms of drug resistance. J. Pathol..

[33] Mansoori B., Mohammadi A., Davudian S., Shirjang S., Baradaran B. (2017). The different mechanisms of cancer drug resistance: A brief review. Adv. Pharm. Bull..

[34] Majidinia M., Mirza-Aghazadeh-Attari M., Rahimi M., Mihanfar A., Karimian A., Safa A., Yousefi B. (2020). Overcoming multidrug resistance in cancer: Recent progress in nanotechnology and new horizons. IUBMB Life.

[35] Dallavalle S., Dobričić V., Lazzarato L., Gazzano E., Machuqueiro M., Pajeva I., Tsakovska I., Zidar N., Fruttero R. (2020). Improvement of conventional anti-cancer drugs as new tools against multidrug resistant tumors. Drug Resist. Updates.

[36] Horsham J.L., Ganda C., Kalinowski F.C., Brown R.A., Epis M.R., Leedman P.J. (2015). MicroRNA-7: A miRNA with expanding roles in development and disease. Int. J. Biochem. Cell Biol..

[37] Zhao J., Tao Y., Zhou Y., Qin N., Chen C., Tian D., Xu L. (2015). MicroRNA-7: A promising new target in cancer therapy. Cancer Cell Int..

[38] Kalinowski F.C., Brown R.A., Ganda C., Giles K.M., Epis M.R., Horsham J., Leedman P.J. (2014). microRNA-7: A tumor suppressor miRNA with therapeutic potential. Int. J. Biochem. Cell Biol..

[39] Zhao J., Zhou Y., Guo M., Yue D., Chen C., Liang G., Xu L. (2020). MicroRNA-7: Expression and function in brain physiological and pathological processes. Cell Biosci..

[40] Horsham J.L., Kalinowski F.C., Epis M.R., Ganda C., Brown R.A.M., Leedman P.J. (2015). Clinical potential of microRNA-7 in cancer. J. Clin. Med..

[41] Mohorianu I., Fowler E.K., Dalmay T., Chapman T. (2018). Control of seminal fluid protein expression via regulatory hubs in *Drosophila melanogaster*. Proc. R. Soc. B Biol. Sci..

[42] Liu K., Feng F., Yang Y., Duan J., Liu H., Yang J., Wu M., Liu C., Chang Y. (2019). High-throughput screening identified miR-7-2-3p and miR-29c-3p as metastasis suppressors in gallbladder carcinoma. J. Gastroenterol..

[43] Wu H., Wei Y., Pan S. (2019). Down-regulation and clinical significance of miR-7-2-3p in papillary thyroid carcinoma with multiple detecting methods. IET Syst. Biol..

[44] Pallarès-Albanell J., Zomeño-Abellán M.T., Escaramís G., Pantano L., Soriano A., Segura M.F., Martí E. (2019). A high-throughput screening identifies MicroRNA inhibitors that influence neuronal maintenance and/or response to oxidative stress. Mol. Ther. Nucleic Acids.

[45] Chakrabarti M., Ray S.K. (2015). Anti-tumor activities of luteolin and silibinin in glioblastoma cells: Overexpression of miR-7-1-3p augmented luteolin and silibinin to inhibit autophagy and induce apoptosis in glioblastoma in vivo. Apoptosis.

[46] Chou Y.-T., Lin H.-H., Lien Y.-C., Wang Y.-H., Hong C.-F., Kao Y.-R., Lin S.-C., Chang Y.-C., Lin S.-Y., Chen S.-J. (2010). EGFR promotes lung tumorigenesis by activating miR-7 through a Ras/ERK/Myc pathway that targets the Ets2 transcriptional repressor ERF. Cancer Res..

[47] Reddy S.D.N., Ohshiro K., Rayala S.K., Kumar R. (2008). MicroRNA-7, a homeobox D10 target, inhibits p21-activated kinase 1 and regulates its functions. Cancer Res..

[48] Ning B.-F., Ding J., Liu J., Yin C., Xu W.-P., Cong W.-M., Zhang Q., Chen F., Han T., Deng X. (2014). Hepatocyte nuclear factor 4α-nuclear factor-κB feedback circuit modulates liver cancer progression. Hepatology.

[49] McInnes N., Sadlon T.J., Brown C.Y., Pederson S., Beyer M., Schultze J.L., McColl S., Goodall G.J., Barry S.C. (2011). FOXP3 and FOXP3-regulated microRNAs suppress SATB1 in breast cancer cells. Oncogene.

[50] Choi D.C., Chae Y.-J., Kabaria S., Chaudhuri A.D., Jain M.R., Li H., Mouradian M.M., Junn E. (2014). MicroRNA-7 protects against 1-methyl-4-phenylpyridinium-induced cell death by targeting RelA. J. Neurosci..

[51] Li M., Pan M., Wang J., You C., Zhao F., Zheng D., Guo M., Xu H., Wu D., Wang L. (2020). miR-7 reduces breast cancer stem cell metastasis via inhibiting RELA to decrease ESAM expression. Mol. Ther. Oncolytics.

[52] Zhao X.-D., Lu Y.-Y., Guo H., Xie H.-H., He L.-J., Shen G.-F., Zhou J.-F., Li T., Hu S.-J., Zhou L. (2015). MicroRNA-7/NF-κB signaling regulatory feedback circuit regulates gastric carcinogenesis. J. Cell Biol..

[53] Duex J.E., Comeau L., Sorkin A., Purow B., Kefas B. (2011). Usp18 regulates epidermal growth factor (EGF) receptor expression and cancer cell survival via MicroRNA-7. J. Biol. Chem..

[54] Choudhury N.R., Alves F.D.L., De Andrés-Aguayo L., Graf T., Cáceres J.F., Rappsilber J., Michlewski G. (2013). Tissue-specific control of brain-enriched miR-7 biogenesis. Genes Dev..

[55] Wang Y., Vogel G., Yu Z., Richard S. (2013). The QKI-5 and QKI-6 RNA binding proteins regulate the expression of MicroRNA 7 in glial cells. Mol. Cell. Biol..

[56] Wu H., Sun S., Tu K., Gao Y., Xie B., Krainer A.R., Zhu J. (2010). A splicing-independent function of SF2/ASF in MicroRNA processing. Mol. Cell.

[57] Mitra A., Pfeifer K., Park K.-S. (2018). Circular RNAs and competing endogenous RNA (ceRNA) networks. Transl. Cancer Res..

[58] Zhao W., Dong M., Pan J., Wang Y., Zhou J., Ma J., Liu S. (2019). Circular RNAs: A novel target among non-coding RNAs with potential roles in malignant tumors (review). Mol. Med. Rep..

[59] Su C., Han Y., Zhang H., Li Y., Yi L., Wang X., Zhou S., Yu D., Song X., Xiao N. (2018). CiRS-7 targeting miR-7 modulates the progression of non-small cell lung cancer in a manner dependent on NF-κB signalling. J. Cell. Mol. Med..

[60] Huang H., Wei L., Qin T., Yang N., Li Z., Xu Z. (2019). Circular RNA ciRS-7 triggers the migration and invasion of esophageal squamous cell carcinoma via miR-7/KLF4 and NF-κB signals. Cancer Biol. Ther..

[61] Han J.-Y., Guo S., Wei N., Xue R., Li W., Dong G., Li J., Tian X., Chen C., Qiu S. (2020). ciRS-7 Promotes the proliferation and migration of papillary thyroid cancer by negatively regulating the miR-7/epidermal growth factor receptor axis. BioMed Res. Int..

[62] Tang W., Ji M., He G., Yang L., Niu Z., Jian M., Wei Y., Ren L., Xu J. (2017). Silencing CDR1as inhibits colorectal cancer progression through regulating microRNA-7. Onco Targets Ther..

[63] Hansen T.B., Jensen T.I., Clausen B.H., Bramsen J.B., Finsen B., Damgaard C.K., Kjems J. (2013). Natural RNA circles function as efficient microRNA sponges. Nature.

[64] Sang Q., Liu X., Wang L., Qi L., Sun W., Wang W., Sun Y., Zhang H. (2018). CircSNCA downregulation by pramipexole treatment mediates cell apoptosis and autophagy in Parkinson’s disease by targeting miR-7. Aging.

[65] Gao D., Qi X., Zhang X., Fang K., Guo Z., Li L. (2019). hsa_circRNA_0006528 as a competing endogenous RNA promotes human breast cancer progression by sponging miR-7-5p and activating the MAPK/ERK signaling pathway. Mol. Carcinog..

[66] Gao D., Zhang X., Liu B., Meng D., Fang K., Guo Z., Li L. (2017). Screening circular RNA related to chemotherapeutic resistance in breast cancer. Epigenomics.

[67] Li G., Huang M., Cai Y., Yang Y., Sun X., Ke Y. (2019). Circ-U2AF1 promotes human glioma via derepressing neuro-oncological ventral antigen 2 by sponging hsa-miR-7-5p. J. Cell. Physiol..

[68] Gao Y., Liu J., Huan J., Che F. (2020). Downregulation of circular RNA hsa_circ_0000735 boosts prostate cancer sensitivity to docetaxel via sponging miR-7. Cancer Cell Int..

[69] Li H., Lan M., Liao X., Tang Z., Yang C. (2020). Circular RNA cir-ITCH promotes osteosarcoma migration and invasion through cir-ITCH/miR-7/EGFR pathway. Technol. Cancer Res. Treat..

[70] Wang Q., Li Z., Hu Y., Zheng W., Tang W., Zhai C., Gu Z., Tao J., Wang H. (2019). Circ-TFCP2L1 promotes the proliferation and migration of triple negative breast cancer through sponging miR-7 by inhibiting PAK1. J. Mammary Gland. Biol. Neoplasia.

[71] Liu L., Yang X., Li N.-F., Lin L., Luo H. (2019). Circ_0015756 promotes proliferation, invasion and migration by microRNA-7-dependent inhibition of FAK in hepatocellular carcinoma. Cell Cycle.

[72] Yao R.-W., Wang Y., Chen L.-L. (2019). Cellular functions of long noncoding RNAs. Nat. Cell Biol..

[73] Zheng Y., Nie P., Xu S. (2020). Long noncoding RNA CASC21 exerts an oncogenic role in colorectal cancer through regulating miR-7-5p/YAP1 axis. Biomed. Pharmacother..

[74] Zheng W., Li H., Zhang H., Zhang C., Zhu Z., Liang H., Zhou Y. (2020). Long noncoding RNA RHPN1-AS1 promotes colorectal cancer progression via targeting miR-7-5p/OGT axis. Cancer Cell Int..

[75] Liu M.-L., Zhang Q., Yuan X., Jin L., Wang L.-L., Fang T.-T., Wang W.-B. (2018). Long noncoding RNA RP4 functions as a competing endogenous RNA through miR-7-5p sponge activity in colorectal cancer. World J. Gastroenterol..

[76] Eckhart L., Lachner J., Tschachler E., Rice R.H. (2020). TINCR is not a non-coding RNA but encodes a protein component of cornified epidermal keratinocytes. Exp. Dermatol..

[77] Song X.-Z., Ren X.-N., Xu X.-J., Ruan X.-X., Wang Y.-L., Yao T.-T. (2020). LncRNA RHPN1-AS1 promotes cell proliferation, migration and invasion through targeting miR-7-5p and activating PI3K/AKT/mTOR pathway in hepatocellular carcinoma. Technol. Cancer Res. Treat..

[78] Hu H., Yang L., Li L., Zeng C. (2018). Long non-coding RNA KCNQ1OT1 modulates oxaliplatin resistance in hepatocellular carcinoma through miR-7-5p/ ABCC1 axis. Biochem. Biophys. Res. Commun..

[79] Yuan C., Luo X., Duan S., Guo L. (2020). Long noncoding RNA LINC00115 promotes breast cancer metastasis by inhibiting miR-7. FEBS Open Bio.

[80] Li D.S., Ainiwaer J.L., Sheyhiding I., Zhang Z., Zhang L.W. (2016). Identification of key long non-coding RNAs as competing endogenous RNAs for miRNA-mRNA in lung adenocarcinoma. Eur. Rev. Pharmacol. Sci..

[81] He H., Dai J., Zhuo R., Zhao J., Wang H., Sun F., Zhu Y., Xu D. (2018). Study on the mechanism behind lncRNA MEG3 affecting clear cell renal cell carcinoma by regulating miR-7/RASL11B signaling. J. Cell. Physiol..

[82] Zhang H., Cai K., Wang J., Wang X., Cheng K., Shi F., Jiang L., Zhang Y., Dou J. (2014). MiR-7, inhibited indirectly by LincRNA HOTAIR, directly inhibits SETDB1 and reverses the EMT of breast cancer stem cells by downregulating the STAT3 pathway. Stem Cells.

[83] Liu X., Fu Q., Li S., Liang N., Li F., Li C., Sui C., Dionigi G., Sun H. (2019). LncRNA FOXD2-AS1 functions as a competing endogenous RNA to regulate TERT expression by sponging miR-7-5p in thyroid cancer. Front. Endocrinol..

[84] Zhang X., Zhao X., Li Y., Zhou Y., Zhang Z. (2019). Long noncoding RNA SOX21-AS1 promotes cervical cancer progression by competitively sponging miR-7/VDAC1. J. Cell. Physiol..

[85] Yang Z., Shi X., Li C., Wang X., Hou K., Li Z., Zhang X., Fan Y., Qu X., Che X. (2018). Long non-coding RNA UCA1 upregulation promotes the migration of hypoxia-resistant gastric cancer cells through the miR-7-5p/EGFR axis. Exp. Cell Res..

[86] Li G., Gao L., Zhao J., Liu D., Li H., Hu M. (2020). LncRNA ANRIL/miR-7-5p/TCF4 axis contributes to the progression of T cell acute lymphoblastic leukemia. Cancer Cell Int..

[87] Kleaveland B., Shi C.Y., Stefano J., Bartel D.P. (2018). A network of noncoding regulatory RNAs acts in the mammalian brain. Cell.

[88] Needhamsen M., White R.B., Giles K.M., Dunlop S.A., Thomas M.G. (2014). Regulation of human PAX6 expression by miR-7. Evol. Bioinform. Online.

[89] Fan Z., Lu M., Qiao C., Zhou Y., Ding J.-H., Hu G. (2016). MicroRNA-7 enhances subventricular zone neurogenesis by inhibiting NLRP3/caspase-1 axis in adult neural stem cells. Mol. Neurobiol..

[90] Hu G., Niu F., Liao K., Periyasamy P., Sil S., Liu J., Dravid S.M., Buch S. (2020). HIV-1 tat-induced astrocytic extracellular vesicle miR-7 impairs synaptic architecture. J. Neuroimmune Pharmacol..

[91] Li S., Lv X., Zhai K., Xu R., Zhang Y., Zhao S., Qin X., Yin L., Lou J. (2016). MicroRNA-7 inhibits neuronal apoptosis in a cellular Parkinson’s disease model by targeting Bax and Sirt2. Am. J. Transl. Res..

[92] Gajda E., Godlewska M., Mariak Z., Nazaruk E., Gawel D. (2020). Combinatory treatment with miR-7-5p and drug-loaded cubosomes effectively impairs cancer cells. Int. J. Mol. Sci..

[93] Visani M., De Biase D., Marucci G., Cerasoli S., Nigrisoli E., Reggiani M.L.B., Albani F., Baruzzi A., Pession A., The PERNO Study Group (2013). Expression of 19 microRNAs in glioblastoma and comparison with other brain neoplasia of grades I–III. Mol. Oncol..

[94] Kumar V., Kumar V., Chaudhary A.K., Coulter D.W., McGuire T., Mahato R.I. (2018). Impact of miRNA-mRNA profiling and their correlation on medulloblastoma tumorigenesis. Mol. Ther. Nucleic Acids.

[95] Chen H., Shalom-Feuerstein R., Riley J., Zhang S.-D., Tucci P., Agostini M., Aberdam D., Knight R.A., Genchi G., Nicotera P. (2010). miR-7 and miR-214 are specifically expressed during neuroblastoma differentiation, cortical development and embryonic stem cells differentiation, and control neurite outgrowth in vitro. Biochem. Biophys. Res. Commun..

[96] Madadi S., Schwarzenbach H., Saidijam M., Mahjub R., Soleimani M. (2019). Potential microRNA-related targets in clearance pathways of amyloid-β: Novel therapeutic approach for the treatment of Alzheimer’s disease. Cell Biosci..

[97] Zhang J., Sun X.-Y., Zhang L.-Y. (2015). MicroRNA-7/Shank3 axis involved in schizophrenia pathogenesis. J. Clin. Neurosci..

[98] Yue D., Zhao J., Chen H., Guo M., Chen C., Zhou Y., Xu L. (2020). MicroRNA-7, synergizes with RORα, negatively controls the pathology of brain tissue inflammation. J. Neuroinflammation.

[99] Zhang X.-D., Fan Q.-Y., Qiu Z., Chen S. (2018). MiR-7 alleviates secondary inflammatory response of microglia caused by cerebral hemorrhage through inhibiting TLR4 expression. Eur. Rev. Med. Pharmacol. Sci..

[100] Correa-Medina M., Bravo-Egana V., Rosero S., Ricordi C., Edlund H., Diez J., Pastori R.L. (2009). MicroRNA miR-7 is preferentially expressed in endocrine cells of the developing and adult human pancreas. Gene Expr. Patterns.

[101] Wang Y., Liu J., Liu C., Naji A., Stoffers D.A. (2012). MicroRNA-7 regulates the mTOR Pathway and proliferation in adult pancreatic β-cells. Diabetes.

[102] Downing S., Zhang F., Chen Z., Tzanakakis E.S. (2019). MicroRNA-7 directly targets Reg1 in pancreatic cells. Am. J. Physiol. Physiol..

[103] Nieto M., Hevia P., Garcia E., Klein D., Alvarez-Cubela S., Bravo-Egana V., Rosero S., Molano R.D., Vargas N., Ricordi C. (2012). Antisense miR-7 impairs insulin expression in developing pancreas and in cultured pancreatic buds. Cell Transplant..

[104] Matarese A., Gambardella J., Lombardi A., Wang X., Santulli G. (2020). miR-7 regulates GLP-1-mediated insulin release by targeting β-arrestin 1. Cells.

[105] Ye Z.-Q., Zou C.-L., Chen H.-B., Jiang M.-J., Mei Z., Gu D.-N. (2020). MicroRNA-7 as a potential biomarker for prognosis in pancreatic cancer. Dis. Markers.

[106] Xia J., Cao T., Ma C., Shi Y., Sun Y., Wang Z.P., Ma J. (2018). miR-7 suppresses tumor progression by directly targeting MAP3K9 in pancreatic cancer. Mol. Ther. Nucleic Acids.

[107] Moazzeni H., Najafi A., Khani M. (2017). Identification of direct target genes of miR-7, miR-9, miR-96, and miR-182 in the human breast cancer cell lines MCF-7 and MDA-MB-231. Mol. Cell. Probes.

[108] Xiao H. (2019). MiR-7-5p suppresses tumor metastasis of non-small cell lung cancer by targeting NOVA2. Cell. Mol. Biol. Lett..

[109] Giles K.M., Brown R.A., Ganda C., Podgorny M.J., Candy P.A., Wintle L.C., Richardson K.L., Kalinowski F.C., Stuart L.M., Epis M.R. (2016). microRNA-7-5p inhibits melanoma cell proliferation and metastasis by suppressing RelA/NF-κB. Oncotarget.

[110] Xu K., Song X., Chen Z., Qin C. (2014). miR-7 inhibits colorectal cancer cell proliferation and induces apoptosis by targeting XRCC2. Onco Targets Ther..

[111] Wu W., Liu S., Liang Y., Zhou Z., Liu X. (2017). MiR-7 inhibits progression of hepatocarcinoma by targeting KLF-4 and promises a novel diagnostic biomarker. Cancer Cell Int..

[112] Liu S., Zhou C., Zhu C., Song Q., Wen M., Liu Y., An H. (2017). Low-expression of miR-7 promotes cell proliferation and exhibits prognostic value in osteosarcoma patients. Int. J. Clin. Exp. Pathol..

[113] Suto T., Yokobori T., Yajima R., Morita H., Fujii T., Yamaguchi S., Altan B., Tsutsumi S., Asao T., Kuwano H. (2014). MicroRNA-7 expression in colorectal cancer is associated with poor prognosis and regulates cetuximab sensitivity via EGFR regulation. Carcinogenesis.

[114] Fan X., Liu M., Tang H., Leng D., Hu S., Lu R., Wan W., Yuan S. (2019). MicroRNA-7 exerts antiangiogenic effect on colorectal cancer via ERK signaling. J. Surg. Res..

[115] Dong M., Xie Y., Xu Y. (2019). miR-7-5p regulates the proliferation and migration of colorectal cancer cells by negatively regulating the expression of Krüppel-like factor 4. Oncol. Lett..

[116] Ling Y., Cao C., Li S., Qiu M., Shen G., Chen Z., Yao F., Chen W. (2019). TRIP6, as a target of miR-7, regulates the proliferation and metastasis of colorectal cancer cells. Biochem. Biophys. Res. Commun..

[117] Lin J., Liu Z., Liao S., Li E., Wu X., Zeng W. (2020). Elevated microRNA-7 inhibits proliferation and tumor angiogenesis and promotes apoptosis of gastric cancer cells via repression of Raf-1. Cell Cycle.

[118] Shi Y., Luo X., Li P., Tan J., Wang X., Xiang T., Ren G. (2015). miR-7-5p suppresses cell proliferation and induces apoptosis of breast cancer cells mainly by targeting REGγ. Cancer Lett..

[119] Kong X., Li G., Yuan Y., He Y., Wu X., Zhang W., Wu Z., Chen T., Wu W., Lobie P.E. (2012). MicroRNA-7 inhibits epithelial-to-mesenchymal transition and metastasis of breast cancer cells via targeting FAK expression. PLoS ONE.

[120] Yin C., Kong W., Jiang J., Xu H., Zhao W. (2018). miR-7-5p inhibits cell migration and invasion in glioblastoma through targeting SATB1. Oncol. Lett..

[121] Liu Z., Liu Y., Li L., Xu Z., Bi B., Wang Y., Li J.Y. (2014). MiR-7-5p is frequently downregulated in glioblastoma microvasculature and inhibits vascular endothelial cell proliferation by targeting RAF1. Tumor Biol..

[122] Babae N., Bourajjaj M., Liu Y., Van Beijnum J.R., Cerisoli F., Scaria P.V., Verheul M., Van Berkel M.P., Pieters E.H.E., Van Haastert R.J. (2014). Systemic miRNA-7 delivery inhibits tumor angiogenesis and growth in murine xenograft glioblastoma. Oncotarget.

[123] Xiong S., Zheng Y., Jiang P., Liu R., Liu X., Chu Y. (2011). MicroRNA-7 inhibits the growth of human non-small cell lung cancer A549 cells through targeting BCL-2. Int. J. Biol. Sci..

[124] Feng Y., Spezia M., Huang S., Yuan C., Zeng Z., Zhang L., Ji X., Liu W., Huang B., Luo W. (2018). Breast cancer development and progression: Risk factors, cancer stem cells, signaling pathways, genomics, and molecular pathogenesis. Genes Dis..

[125] Hashmi A.A., Hashmi K.A., Irfan M., Khan S.M., Edhi M.M., Ali J.P., Hashmi S.K., Asif H., Faridi N., Khan A. (2019). Ki67 index in intrinsic breast cancer subtypes and its association with prognostic parameters. BMC Res. Notes.

[126] Hoeferlin L.A., Chalfant C.E., Park M.A. (2013). Challenges in the treatment of triple negative and HER2-overexpressing breast cancer. J. Surg. Sci..

[127] Zhao S., Zuo W.-J., Shao Z.-M., Jiang Y.-Z. (2020). Molecular subtypes and precision treatment of triple-negative breast cancer. Ann. Transl. Med..

[128] Musgrove E.A., Sutherland R.L. (2009). Biological determinants of endocrine resistance in breast cancer. Nat. Rev. Cancer.

[129] Gonzalez-Angulo A.M., Morales-Vasquez F., Hortobagyi G.N. (2007). Overview of resistance to systemic therapy in patients with breast cancer. Adv. Exp. Med. Biol..

[130] Yang W., Yang X., Wang X., Gu J., Zhou D., Wang Y., Yin B., Guo J., Zhou M. (2019). Silencing CDR1 as enhances the sensitivity of breast cancer cells to drug resistance by acting as a miR-7 sponge to down-regulate REGγ. J. Cell. Mol. Med..

[131] Pogribny I.P., Filkowski J.N., Tryndyak V.P., Golubov A., Shpyleva S.I., Kovalchuk O. (2010). Alterations of microRNAs and their targets are associated with acquired resistance of MCF-7 breast cancer cells to cisplatin. Int. J. Cancer.

[132] Huang Q., Wu Y.-Y., Xing S.-J., Yu Z.-W. (2019). Effect of miR-7 on resistance of breast cancer cells to adriamycin via regulating EGFR/PI3K signaling pathway. Eur. Rev. Med. Pharmacol. Sci..

[133] Uhr K., Sieuwerts A.M., De Weerd V., Smid M., Hammerl D., Foekens J.A., Martens J.W.M. (2018). Association of microRNA-7 and its binding partner CDR1-AS with the prognosis and prediction of 1st-line tamoxifen therapy in breast cancer. Sci. Rep..

[134] Raychaudhuri M., Bronger H., Buchner T., Kiechle M., Weichert W., Avril S. (2017). MicroRNAs miR-7 and miR-340 predict response to neoadjuvant chemotherapy in breast cancer. Breast Cancer Res. Treat..

[135] Hong T., Ding J., Li W. (2019). miR-7 reverses breast cancer resistance to chemotherapy by targeting MRP1 And BCL2. Onco Targets Ther..

[136] Huynh F.C., Jones F.E. (2014). MicroRNA-7 inhibits multiple oncogenic pathways to suppress HER2Δ16 mediated breast tumorigenesis and reverse trastuzumab resistance. PLoS ONE.

[137] Hsiao Y.-C., Yeh M.-H., Chen Y.-J., Liu J.-F., Tang C.-H., Huang W.-C. (2015). Lapatinib increases motility of triple-negative breast cancer cells by decreasing miRNA-7 and inducing Raf-1/MAPK-dependent interleukin-6. Oncotarget.

[138] Zappa C., Mousa S.A. (2016). Non-small cell lung cancer: Current treatment and future advances. Transl. Lung Cancer Res..

[139] Yang S.-R., Schultheis A.M., Yu H., Mandelker D., Ladanyi M., Büttner R. (2020). Precision medicine in non-small cell lung cancer: Current applications and future directions. Semin. Cancer Biol..

[140] Tsoukalas N., Aravantinou-Fatorou E., Baxevanos P., Tolia M., Tsapakidis K., Galanopoulos M., Liontos M., Kyrgias G. (2018). Advanced small cell lung cancer (SCLC): New challenges and new expectations. Ann. Transl. Med..

[141] Ge X., Zheng L., Huang M., Wang Y., Bi F. (2014). MicroRNA expression profiles associated with acquired gefitinib-resistance in human lung adenocarcinoma cells. Mol. Med. Rep..

[142] Zhao J.-G., Men W.-F., Tang J. (2015). MicroRNA-7 enhances cytotoxicity induced by gefitinib in non-small cell lung cancer via inhibiting the EGFR and IGF1R signalling pathways. Współczesna Onkol..

[143] Mou K., Gu W., Gu C., Zhang J., Qwang W., Ren G., Tian J. (2016). Relationship between miR-7 expression and treatment outcomes with gefitinib in non-small cell lung cancer. Oncol. Lett..

[144] Cheng M.-W., Shen Z.-T., Hu G.-Y., Luo L.-G. (2017). Prognostic significance of microRNA-7 and its roles in the regulation of cisplatin resistance in lung adenocarcinoma. Cell. Physiol. Biochem..

[145] He X., Li C., Wu X., Yang G. (2015). Docetaxel inhibits the proliferation of non-small-cell lung cancer cells via upregulation of microRNA-7 expression. Int. J. Clin. Exp. Pathol..

[146] Liu R., Liu X., Zheng Y., Gu J., Xiong S., Jiang P., Jiang X., Huang E., Yang Y., Ge D. (2014). MicroRNA-7 sensitizes non-small cell lung cancer cells to paclitaxel. Oncol. Lett..

[147] Liu H., Wu X., Huang J., Peng J., Guo L. (2015). miR-7 modulates chemoresistance of small cell lung cancer by repressing MRP1/ABCC1. Int. J. Exp. Pathol..

[148] Guo L., Liu Y., Bai Y., Sun Y., Xiao F., Guo Y. (2010). Gene expression profiling of drug-resistant small cell lung cancer cells by combining microRNA and cDNA expression analysis. Eur. J. Cancer.

[149] Liu H., Huang J., Peng J., Wu X., Zhang Y., Zhu W., Guo L. (2015). Upregulation of the inwardly rectifying potassium channel Kir2.1 (KCNJ2) modulates multidrug resistance of small-cell lung cancer under the regulation of miR-7 and the Ras/MAPK pathway. Mol. Cancer.

[150] Lai J., Yang H., Zhu Y., Ruan M., Huang Y., Zhang Q. (2019). MiR-7-5p-mediated downregulation of PARP1 impacts DNA homologous recombination repair and resistance to doxorubicin in small cell lung cancer. BMC Cancer.

[151] Taylor O.G., Brzozowski J.S., Skelding K.A. (2019). Glioblastoma multiforme: An overview of emerging therapeutic targets. Front. Oncol..

[152] Zhang H., Wang R., Yu Y., Liu J., Luo T., Fan F. (2019). Glioblastoma treatment modalities besides surgery. J. Cancer.

[153] Jia B., Liu W., Gu J., Wang J., Lv W., Zhang W., Hao Q., Pang Z., Mu N., Zhang W. (2019). MiR-7-5p suppresses stemness and enhances temozolomide sensitivity of drug-resistant glioblastoma cells by targeting Yin Yang 1. Exp. Cell Res..

[154] Balogh J., Victor D., Asham E.H., Burroughs S.G., Boktour M., Saharia A., Li X., Ghobrial R.M., Monsour H.P. (2016). Hepatocellular carcinoma: A review. J. Hepatocell. Carcinoma.

[155] Kabir T.D., Ganda C., Brown R.M., Beveridge D.J., Richardson K.L., Chaturvedi V., Candy P., Epis M., Wintle L., Kalinowski F. (2018). A microRNA-7/growth arrest specific 6/TYRO3 axis regulates the growth and invasiveness of sorafenib-resistant cells in human hepatocellular carcinoma. Hepatology.

[156] Torre L.A., Trabert B., DeSantis C.E., Mph K.D.M., Samimi G., Runowicz C.D., Gaudet M.M., Jemal A., Siegel R.L. (2018). Ovarian cancer statistics. CA Cancer J. Clin..

[157] Cui X., Sun Y., Shen M., Song K., Yin X., Di W., Duan Y. (2018). Enhanced chemotherapeutic efficacy of paclitaxel nanoparticles co-delivered with MicroRNA-7 by inhibiting paclitaxel-induced EGFR/ERK pathway activation for ovarian cancer therapy. ACS Appl. Mater. Interfaces.

[158] Vera O., Jimenez J., Pernia O., Rodríguez-Antolín C., Rodriguez C., Cabo F.S., Soto J., Rosas R., Lopez-Magallon S., Rodriguez I.E. (2017). DNA methylation of miR-7 is a mechanism involved in platinum response through MAFG overexpression in cancer cells. Theranostics.

[159] Xu N., Lian Y.-J., Dai X., Wang Y.-J. (2017). miR-7 increases cisplatin sensitivity of gastric cancer cells through suppressing mTOR. Technol. Cancer Res. Treat..

[160] Yang F., Guo L., Cao Y., Li S., Li J., Liu M. (2018). MicroRNA-7-5p promotes cisplatin resistance of cervical cancer cells and modulation of cellular energy homeostasis by regulating the expression of the PARP-1 and BCL2 genes. Med. Sci. Monit..

[161] Jiang M.-J., Dai J.-J., Gu D.-N., Huang Q., Tian L. (2017). MicroRNA-7 inhibits cell proliferation of chronic myeloid leukemia and sensitizes it to imatinib in vitro. Biochem. Biophys. Res. Commun..

[162] Sun X., Li J., Sun Y., Zhang Y., Dong L., Shen C., Yang L., Yang M., Li Y., Shen G. (2016). miR-7 reverses the resistance to BRAFi in melanoma by targeting EGFR/IGF-1R/CRAF and inhibiting the MAPK and PI3K/AKT signaling pathways. Oncotarget.

[163] Segal M., Slack F.J. (2020). Challenges identifying efficacious miRNA therapeutics for cancer. Expert Opin. Drug Discov..

[164] Torres A.G., Gait M.J. (2012). Exploiting cell surface thiols to enhance cellular uptake. Trends Biotechnol..

[165] Abumanhal-Masarweh H., Da Silva D., Poley M., Zinger A., Goldman E., Krinsky N., Kleiner R., Shenbach G., Schroeder J.E., Shklover J. (2019). Tailoring the lipid composition of nanoparticles modulates their cellular uptake and affects the viability of triple negative breast cancer cells. J. Control. Release.

[166] Dziawer Ł., Majkowska-Pilip A., Gaweł D., Godlewska M., Pruszyński M., Jastrzębski J., Wąs B., Bilewicz A. (2019). Trastuzumab-modified gold nanoparticles labeled with 211At as a prospective tool for local treatment of HER2-positive breast cancer. Nanomaterials.

[167] Kettler K., Veltman K., Van De Meent D., Van Wezel A., Hendriks A.J. (2014). Cellular uptake of nanoparticles as determined by particle properties, experimental conditions, and cell type. Environ. Toxicol. Chem..

[168] Huang C.-K., Kafert-Kasting S., Thum T. (2020). Preclinical and clinical development of noncoding RNA therapeutics for cardiovascular disease. Circ. Res..

[169] Van der Ree M.H., van der Meer A.J., van Nuenen A.C., de Bruijne J., Ottosen S., Janssen H.L., Kootstra N.A., Reesink H.W. (2016). Miravirsen dosing in chronic hepatitis C patients results in decreased microRNA-122 levels without affecting other microRNAs in plasma. Aliment. Pharmacol. Ther..

